# The role of autophagy in brain health and disease: Insights into exosome and autophagy interactions

**DOI:** 10.1016/j.heliyon.2024.e38959

**Published:** 2024-10-04

**Authors:** Hai-Dong Wang, Chao-Liang Lv, Lei Feng, Jin-Xiu Guo, Shi-Yuan Zhao, Pei Jiang

**Affiliations:** a Department of Pharmacy, The Affiliated Lianyungang Hospital of Xuzhou Medical University/Nanjing Medical University Kangda College First Affiliated Hospital/The First People's Hospital of Lianyungang, Lianyungang, 222000, China; b Department of Spine Surgery, Jining First People's Hospital, Shandong First Medical University, Jining, 272000, China; c Department of Neurosurgery, Jining First People's Hospital, Shandong First Medical University, Jining, 272000, China; d Translational Pharmaceutical Laboratory, Jining First People's Hospital, Shandong First Medical University, Jining, 272000, China; e Institute of Translational Pharmacy, Jining Medical Research Academy, Jining, 272000, China

**Keywords:** Exosomes, Autophagy, Brain, Nervous system, Brain disease

## Abstract

Effective management of cellular components is essential for maintaining brain health, and studies have identified several crucial biological processes in the brain. Among these, autophagy and the role of exosomes in cellular communication are critical for brain health and disease. The interaction between autophagy and exosomes in the nervous system, as well as their contributions to brain damage, have garnered significant attention. This review summarizes that exosomes and their cargoes have been implicated in the autophagy process in the pathophysiology of nervous system diseases. Furthermore, the onset and progression of neurological disorders may be affected by autophagy regulation of the secretion and release of exosomes. These findings may provide new insights into the potential mechanism by which autophagy mediates different exosome secretion and release, as well as the valuable biomedical applications of exosomes in the prevention and treatment of various brain diseases by targeting autophagy.

## 1 Introduction

An appropriate equilibrium between protein synthesis and proteolysis is vital for upholding the functions of cells and tissues within an organism. The brain serves as the centre of the human nervous system, and maintaining its health is essential for human well-being and daily functioning. Typically, an average adult human brain comprises approximately one trillion glial cells and one hundred billion neurons. Despite constituting roughly 2 %–3 % of an individual's total body mass, the human brain is accountable for as much as half of the body's overall consumption of glucose and oxygen, generating more free waste than other organs. Therefore, the brain is more susceptible to neurodegenerative and other nervous system diseases owing to its inherent features [[1], [2], [3], [4]]. Effective management of cellular components, such as the removal of aggregated proteins and malfunctioning organelles and the recycling of cellular elements, is essential for maintaining long-term brain health. Recent research has shown several regulatory mechanisms operating in the brain. Among the many well-studied pathways for the degradation of large targets, including protein aggregates and malfunctioning organelles, autophagy stands out as a key intracellular process for maintaining cellular homeostasis [[5], [6], [7]]. Exosomes, a subset of extracellular vesicles (EVs), have been revealed to potentially function as messengers between cells by transporting DNA and proteins [8]. Exosomes are released in the brain by several neurocytes, including neurons, microglia, and astrocytes, each possessing unique characteristics, regulatory abilities, and functional properties [9].

In this review, we first elucidate the vital functions of autophagy in the brain and then discuss the potential fundamental significance of exosomal influence on brain function. In addition, we highlight the cross-regulation of autophagy and exosomes in the brain, including the impact of exosomes on autophagy and the modulation of exosome secretion and release by autophagy in brain diseases. This review attempts to survey the cross-regulation function of exosomes and autophagy in the development and progression of brain function.

## 2 Autophagy

Heterophagy refers to the process by which cells engulf and degrade external substances, such as bacteria and dead cells, through endocytosis, leading to their breakdown within lysosomes [10,11]. In contrast, autophagy involves the degradation of the cell's own components, such as damaged organelles, within lysosomes. The term “autophagy,” which combines the Greek meanings for “self” and “eating,” is sometimes interpreted as a self-sacrificing mechanism. The primary difference between heterophagy and autophagy lies in the origin of the material being degraded—external in heterophagy and internal in autophagy [12,13]. Autophagy is a profoundly evolutionarily conserved cellular catabolic process that facilitates the clearance of numerous cytoplasmic components. This process is regulated by endocytosis and plays a vital role in maintaining cellular homeostasis and metabolic balance [14,15]. Under normal physiological conditions, autophagy is essential for preserving cellular homeostasis and ensuring cellular health. An imbalance in autophagy may result in various diseases, whereas excessive autophagy could culminate in programmed cell death [15,16].

During autophagy, the autophagosome forms around cargo and fuses with lysosomes, resulting in the degradation of the cargo. Three basic types of autophagy can be identified based on the processes used to shuttle cargo to the lysosomes**:** microautophagy, chaperone-mediated autophagy (CMA), and macroautophagy [15,17]. During microautophagy, cargo is directly taken up by the vacuole, which subsequently delivers the cargo to the lysosome for degradation [18,19]. During CMA, a selective group of cargo is identified by the chaperone protein heat shock protein 70 complex (Hsc70) containing a CMA-targeting motif. It is then transported to the surface of the lysosome, where it interfaces with a lysosomal transmembrane protein called LAMP2A, thereby serving as the cargo receptor for CMA. Cargo proteins are transported into the lysosome and degraded by LAMP2A after undergoing a sequence of complex and sophisticated assembly and disassembly mechanisms [20,21]. CMA is a tightly regulated degradation process that involves Hsc70 and the multimerization of LAMP2A. Moreover, CMA is known to bind to substrates consisting of proteins that include a pentapeptide (KFERQ-like motif) [22].

The primary type of autophagy is macroautophagy, distinguished by its ability to engulf cargo via the production of double-membrane vesicles called autophagosomes. These autophagosomes traverse through multiple maturation stages before finally fusing with lysosomes and discharging their payload for degradation [23]. This research will refer to macroautophagy, the form of autophagy that has garnered the most attention, simply as autophagy.

There is a very intricate and multistage mechanism for autophagy in cells, which is usually regulated by a set of autophagy-related genes (Atgs). The two principal proteins upstream of autophagy in mammalian cells are the target of rapamycin complex 1 (mTORC1) and AMP-activated protein kinase (AMPK) [24]. Simply put, the mTOR pathway acts as a negative regulator of autophagy, and stimulation of the mTOR pathway inhibits autophagy. Conversely, the phosphorylation of AMPK potentially inhibits the activation of mTOR; thus, the activation of AMPK may increase the rate of autophagy [25,26]. In particular, over 40 Atg genes have been identified, many of which encode proteins that are conserved in mammals. Analyses of Atg gene knockout mice have contributed to understanding the physiological roles of autophagy in vivo. In mammals, approximately 20 core autophagy-related genes are involved in the formation of autophagosomes. To date, 14 of these genes have been knocked out in mice. Akiko Kuma and colleagues [27]summarized that some of these Atg knockout mice die in utero or within one day of birth, while others show no apparent abnormalities and survive normally, providing excellent experimental models for studying autophagy. Table 1 summarizes commonly used Atg gene knockout mouse models and their outcomes in autophagy research.

**Table 1:** Summarization of studies on Atg genes KO in mice models.

KO Atg gene	Disease models	Physiological effect	Finding	Reference	
Atg5	Alzheimer disease	Inhibiting postnatal neurogenesis	ATG5 supports neural stem cells health and may prevent neurodegeneration	(Tang et al., 2024)	[226]
	Male reproduction	Atg 5 induces autophagy by mediating multiple signals to maintain normal developmental processes	TG5 is essential for male fertility and is involved in various aspects of spermiogenesis	(Huang et al., 2021)	[227]
	Metabolic liver disease	Deletion of Atg5 in mice inhibit liver tumorigenesis but increase mortality	Atg5 accompanied with p62/Sqstm1, regulate liver cell repopulation, ductular reaction and metabolic reprogramming in liver tumorigenesis.	(Chao et al., 2022)	[228]
	Alzheimer's disease	Postmortem decrease in S403-phos-p62 immunoreactivity,	Post-mortem changes should be considered when interpreting human data	(Kurosawa et al., 2016)	[229]
Atg4b	–	Atg4b (−/−) mice produced by breeding heterozygous parents are produced at a slightly lower than expected ratio to heterozygous	Atg4 homologs have tissue-specific functions beyond redundancy.	(Read et al., 2011)	[230]
	Metabolomic Alterations	Impaired autophagy results in highly tissue-dependent alterations that are more accentuated in the skeletal muscle and plasma.	Impaired autophagy may affect the metabolism of several tissues in mammals.	Martínez-García et al., 2021)	[231]
Atg14	Systemic and hepatic inflammation	Developed hypertrophic cardiomyopathy	Tissue-specific differences between skeletal and cardiac muscles on core autophagy proteins	(Li et al., 2021)	[232]
Atg7	Acute kidney injury	Autophagy deficiency induced after AKI suppressed the pro-fibrotic phenotype in tubular cells and reduced fibrosis.	Persistent autophagy after AKI induces pro-fibrotic phenotype transformation in tubular cells leading to the expression and secretion of FGF2	(Livingston et al., 2023)	[233]
	Hippocampal neurogenesis and cognitive deficits	Atg7 conditional knockout mice have an intact number of NSCs and neurogenesis level under chronic restraint stress and are resilient to stress- or corticosterone-induced cognitive and mood deficits	Autophagic cell death is biologically important in a mammalian system in vivo	(Jung et al., 2020)	[234]
	Nonalcoholic steatohepatitis	Adipocyte-specific Atg7 KO decrease serum free fatty acid levels and ameliorate HFD-induced steatosis, liver inflammation, and fibrosis	Autophagy inhibition in white adipose tissues ameliorates the liver pathology of NAFLD via adipose-liver crosstalk.	(Sakane et al., 2021)	[235]
	Hypertensive disorder	Atg7 conditional knockout (cKO) placentas are significantly smaller than controls in the spongiotrophoblast layer	Placental autophagy is required for normal placentation.	(Aoki et al., 2018)	[236]
	Thrombosis	Loss of endothelial ATG7 attenuates thrombosis and reduces the expression of TF. tissue factor	Endothelial ATG7 is a critical and previously unrecognized target for modulating the susceptibility to thrombosis.	(Yau et al., 2017)	[237]
	Fibrosis	Atg7 deficient mice have increased fibrosis marker (COL1A1, CTGF, TGF-β1, and α-SMA) levels	Atg7 and autophagy-related mechanisms confer radioprotection	(Chen et al., 2023)	[238]
	Myocardial ischemia/reperfusion injury.	Loss of ATG7 abolish the beneficial effects on mitochondrial homeostasis.	Autophagic flux is a sufficient and essential process to mitigate myocardial reperfusion injury by maintaining mitochondrial homeostasis	(He et al., 2022)	[239]
Atg2b	Myeloproliferative neoplasm	Knockout of Atg2b or Gskip alone do not show any hematopoietic abnormality	Atg2b and Gskip play a synergistic effect in maintaining the pool size of hematopoietic stem cells.	(Sakai et al., 2022)	[240]
Becn1	Non-small cell lung cancers	Knockout of Becn1 leads to increase senescence and reduce autophagy.	USP5-Beclin 1 axis is pivotal in overriding intrinsic p53-dependent senescence in Kras-driven lung cancer development	(Li et al., 2022)	[241]
	Traumatic brain injury.	Knockout of the essential autophagy gene Becn1 led to overall increase in neuroinflammation after TBI.	Inhibition of autophagy in microglia and infiltrating macrophages contributes to excessive neuroinflammation following brain injury	(Hegdekar et al., 2023)	[242]
	Fertility	Beclin 1 (Becn1) knockout mice are generated and are conducted to determine the role of Becn1 in spermatogenesis and fertility of mice.	Becn1 is essential for fertility and spermiogenesis in mice.	(Ke et al., 2024)	[243]
	Bronchopulmonary dysplasia	Becn1 ± mice are more susceptible to neonatal hyperoxia-induced lung injury	Preservation of autophagic activity may be an effective protective strategy in Bronchopulmonary dysplasia	(Zhang et al., 2020)	[244]
Ulk1	Neointima formation.	Ulk1 deletion inhibit autophagic degradation of histone acetyltransferase protein	Ulk1 deletion inhibits neointima formation by reducing autophagic degradation of KAT2A and increasing TUBA acetylation in VSMCs.	(Ouyang et al., 2021)	[245]

**Table 2:** Summary of exosomes regulate autophagy in some brain diseases.

Diseases	Exosome derivation	Contents	Autophagy target	Autophagy function	Other physiological function	Reference	
Ischemic stroke	Astrocyte	–	Decrease LC3-II/LC3-I Decrease Beclin-1 level	Increase autophagy	Inhibited neurons apoptosis Ameliorate neuronal damage	(Pei et al., 2019)	[188]
Cerebral ischemia stroke	Adipose-derived grafted mesenchymal stem cells	miR-25-3p	Reduce LC3-II accumulation Reduce abundance of p53	Inhibit autophagy	Neuroprotection Enhanced neurological recovery	(Kuang et al., 2020)	[192]
Ischemic stroke	Ischemic-preconditioned astrocyte	circular RNA circSHOC2	Reduce LC3-II/LC3-I Reduce Beclin-1 Increase the expression level of p62	Inhibit autophagy	Inhibit neuronal death and neuronal apoptosis Ameliorate neuronal damage acting on the miR-7670-3p/SIRT1 axis	(Chen et al., 2020b)	[187]
Ischemic stroke injury	Astrocyte	Nicotinamide phosphoribosyltransferase	Promote the formation of autophagosomes Increase LC3 II/I Promote the production of neuronal autophagy plaques Regulate AMPK/mTOR	Enhance autophagy	Neuroprotective effects Promoted neurofunctional recovery	(Deng et al., 2022)	[194]
Ischemic stroke	Healthy Serum	–	Reduce LC3B-II/LC3B-I Increase the SQSTM1/p62 expression	Reduce autophagy	Reduce infarct volume Improve stroke outcome Reduce the permeability of BBB Reduce endothelial cell apoptosis	(Huang et al., 2022)	[134]
Acute ischemic stroke	Adipose-derived stem cells	miR-30d-5p	Suppress the formation of autophagy plaques Reduce Beclin-1, Atg-5 and LC-3 Increase P62 expression	Inhibit autophagy	Suppress the inflammatory response Promote M2 microglial/macrophage polarization Inhibiting microglial polarization to M1	(Jiang et al., 2018)	[191]
Heat stroke	Microglial	miR-155	Inhibit the activity of mTOR signaling	Induce autophagy		(Li et al., 2022b)	[195]
Ischemic Stroke	Peripheral blood	hyaluronan-binding protein 2	Activate the PAR1/PI3K/AKT/mTOR pathway	Reduce autophagy	Aggravate neuroinflammatory injury	(Luo et al., 2023)	[196]
Ischemic stroke	Astrocyte	miR-190b	Decrease levels of LC3-II/I ratio, Beclin-1 and Atg7 levels Increased P62 level Suppress the protein levels of cleaved caspase-3 and Bax, Promote Bcl-2 expression	Inhibit autophagy	Attenuated neuronal apoptosis	(Pei et al., 2020)	[189]
Cerebral ischemia-reperfusion injury	Bone marrow stromal cell	miR-133a-3p	Upregulate the expressions of p-Akt and p-mTOR	Inhibit autophagy	Inhibit apoptosis	(Yang et al., 2023)	[197]
Cerebral Ischemia/Reperfusion Injury	Bone Marrow Mesenchymal Stem Cells	Long Non-coding RNA KLF3 antisense RNA 1	Regulate Sirt1-mediated Beclin1 deacetylation.	Promote autophagy		(Xu et al., 2023)	[198]
Repetitive traumatic brain injury	BV2 microglia	miR-124-3p	Upregulate FIP200	Inhibit autophagy	Protect against nerve injury	(Li et al., 2019a)	[202]
Traumatic brain injury	Microglia	miR-124-3p	Suppress mTOR signaling	Induce autophagy		(Zhao et al., 2022)	[203]
Traumatic brain injury	neuron	miR-21-5p	Suppress Rab11a	Inhibit autophagy		(Li et al., 2019b)	[204]
Brain injury	Bone marrow stromal cells	miR-32	Regulate disabled homolog 2-interacting protein	Induce autophagy		(Yuan et al., 2020)	[206]
Glioblastoma stem cell	Glioblastoma -derived stem cells		Activate AMPK/ULK1 pathway	Mediate autophagy		(Zheng et al., 2021)	[218]
Alzheimer's disease	M2 microglia		Improve PINK1/Parkin pathway Decrease beclin1, LC3II, PINK1	Ameliorate autophagy	Increased cell viability Restore the destruction of mitochondrial membrane potential Reduce the accumulation of reactive oxygen Neuroprotective	(Li et al., 2022a)	[169]
Parkinson's disease	Mesenchymal stem cells			Induce autophagy	Augment stemness	(Shin et al., 2022)	[178]
Parkinson's disease	Adipose-derived stem cell	miR-188-3p	Inhibit the LC3B expression	Suppress autophagy	Inhibit inflammasomes	(Li et al., 2021)	[179]
Parkinson's disease	Human umbilical cord mesenchymal stem cells			Induce autophagy	Promoted cells to proliferate Inhibited apoptosis Reduced dopaminergic neuron loss Upregulate the levels of dopamine and its metabolites	(Chen et al., 2020a)	[180]

**Fig. 1:**
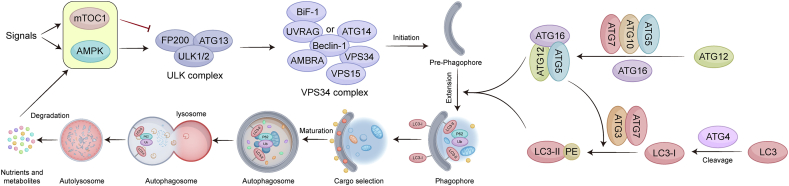
The brief molecular mechanisms of the autophagy signaling pathway. Internal and external signals stimulate the mTOR and AMPK. mTOR inhibits the autophagy initiation complex, ULK complex, consisting of ULK1/2, ATG13, FIP200 and ATG 101a. Additionally, AMPK activation leads to ULK1 phosphorylation, promoting the assembly of the ULK complex. The ULK complex further phosphorylates Beclin1, promoting formation of the VPS34 complex, which initiates autophagy of pre-phagophore. The isolation membrane, or phagophore, is promoted by a series of enzymatic reactions involving ATG proteins and incorporation of ATG homologs (for example, LC3) into the growing membrane. After cargo selection, the maturated autophagosome fuse with lysosome to degrade the cargos by acid hydrolases in lysosomes and then released the nutrients and metabolites to the cytoplasm.

In the presence of reactive oxygen species (ROS), hypoxia, nutrient and energy deprivation, and other forms of deprivation, AMPK may be activated. This activation subsequently triggers autophagy, either directly or indirectly, by inhibiting mTORC1 [28,29]. The inhibition of mTORC results in the phosphorylation of UNC51-like kinase 1 (ULK1), thereby facilitating the formation of a ULK1 complex consisting of ULK1, autophagy-related gene 13 (Atg13), Atg101, and RB1-inducible coiled-coil protein 1 (FIP200). The initiation of phagophore nucleation occurs when this complex phosphorylates Beclin1, a crucial component of the Beclin1-VPS34-Atg14 complex. The Atg12/Atg5-Atg16 complex subsequently mediates autophagosome elongation, which involves the integration of two ubiquitin-like conjugate mechanisms [[30], [31], [32]]. This complex facilitates the conjugation of Atg8 proteins, including gamma-aminobutyric acid receptor-associated proteins (GABARAPs) and microtubule-associated protein 1 light chain 3 (LC3), to membrane-resident phosphatidylethanolamine (PE) with the assistance of Atg3. LC3 exists in two forms: the soluble LC3-I and the insoluble LC3-II, which are stably attached to the membrane of autophagosomes. Both GABARAPs and LC3 are members of the Atg8 protein family and play crucial roles in autophagy. This process results in the formation of lipidated structures capable of binding to membranes. The subsequent sealing of these membranes results in the production of autophagosomes [33,34]. Finally, upon the removal of the Atg proteins, autophagosomes fuse with lysosomes. Within the lysosomes, the cargo is degraded by acid hydrolases, releasing the salvaged nutrients into the cytoplasm (Fig. 1) [35,36].

The human body is made up of a variety of essential organs, with the brain being one of the largest and most complicated of these organs. For the brain to remain healthy and effective for a lifetime, its cells and tissues must efficiently remove aggregated and/or damaged proteins and organelles from their environment. The regulation of autophagy is essential for maintaining homeostasis in the brain and ensuring its continued existence.

### 2.1 Neural autophagy

Neurons play an essential role in the process of brain development as well as its functioning, both of which are primarily characterized by high energy requirements to sustain vast physiological activities that are responsible for sensory perception, thinking, and behavior [37]. Action potentials from neurons may travel at speeds of up to 50–100 impulses per second and be maintained for decades or even centuries [38]. Like other cells, neurons must deal with the issue of aggregated proteins and damaged or aged organelles, which must be removed for proper neuronal growth and long-term health. Nevertheless, neurons are long-lived post-mitotic cells that are incapable of using cell division to degrade damaged or accumulating proteins and organelles. In addition, since neurons have to fulfill such high metabolic demands to accomplish the functions of interpreting and conveying information, they are especially vulnerable to being damaged by oxidative stress. Additionally, the majority of these demands are directed toward the isolated regions, which are the vulnerable regions of neurons that bear the brunt of the stress [39,40]. The importance of autophagy for neurons has been identified in several aspects.

#### 2.1.1 Spatial organization of neuronal autophagy

**Fig. 2:**
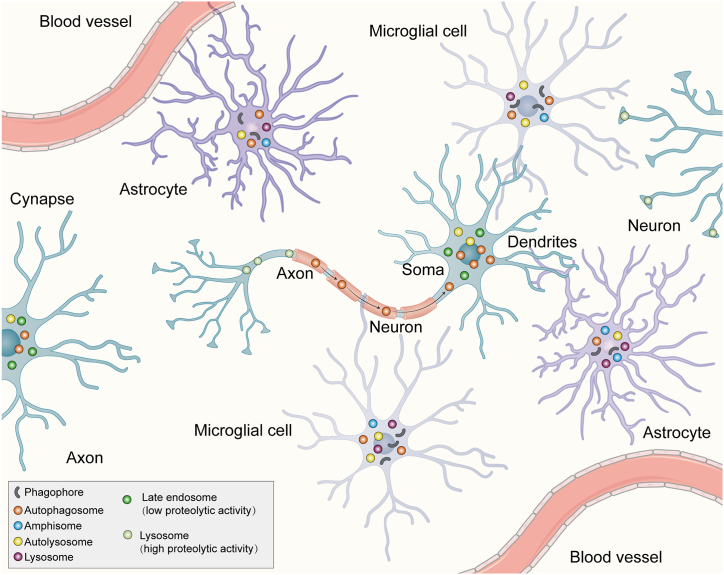
Autophagy in cells of brain. Schematic depicting autophagy in neurons, astrocytes, microglia and neurovascular. In neurons, autophagosomes can form locally within the soma or originate in the distal axon and then travel to the soma. In latter case, autophagosomes fuse with late endosomes in the distal axon following formation, then travel toward the proximal axon and soma and can migrate into dendrites to facilitate post-synaptic functions.

Neurons possess three compartments according to different functions: a soma, the central processing unit of a neuron; an axon, which generates and transmits action potentials; and dendrites containing dendritic spines that collect signals from other neurons [41,42]. Considering the special spatial challenges, the autophagy of neurons has unique spatial features and studies including live-cell imaging of GFP-LC3, which is the classic biomarker for the autophagosome, provides an in-depth comprehension of the characteristics of autophagosome movement in neurons (Fig. 2).

**Fig. 3:**
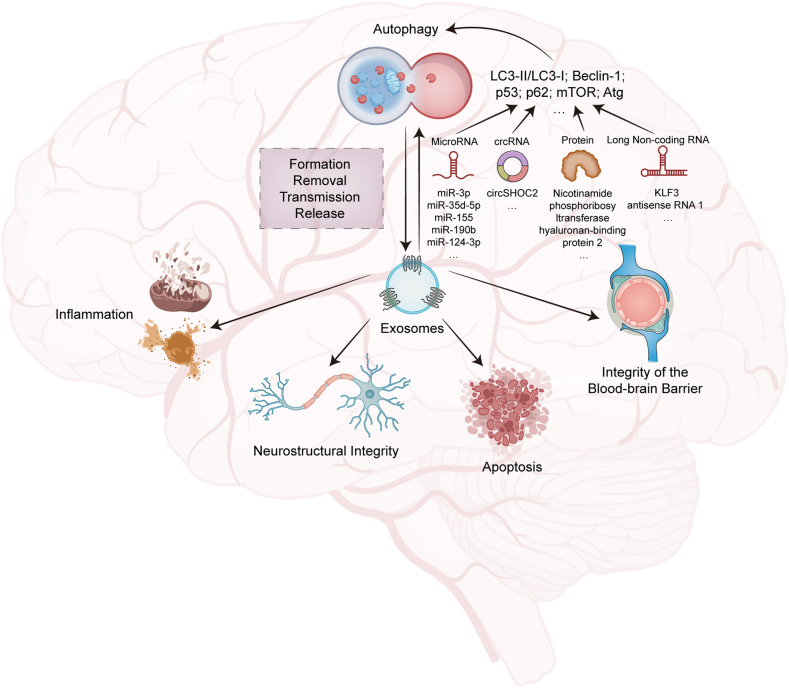
Exosomes affect brain function and interact with autophagy Exosomes can regulate inflammation, affect neurostructural integrity affect the integrity of the blood-brain barrier and inhibit apoptosis in brain. There exists cross-regulation of exosomes and autophagy in brain. Various exosomal contents or composition (MicroRNA, crcRNA, protein, Long non-coding RNA and et al.) can act on different autophagy factors (LC3, Beclin-1, p53, p62 and et al.), inducing or inhibiting autophagy process. Autophagy can affect the formation, removal, transmission and release of exosomes in the brain.

After the autophagosomes have completed their initial production, they are involved in stable retrograde transportation down the axon towards the soma. This retrograde transport requires the dynein and kinesin proteins found in neuronal autophagosomes. Following the completion of the first stage of their journey, the budding autophagosomes begin to merge with late endosomes and/or lysosomes after exiting the distal axon [43]. After which, the transport switches to strongly processive retrograde trafficking toward the cell body, which is regulated by dynein and modulated by its scaffolding proteins JIP1, huntingtin, and HAP1 (huntingtin-associated protein 1) [44]. Therefore, axonal autophagy is a vectorial pathway that delivers cargo long-distance from the distal axon to the soma at a steady state. In the course of autophagosomes' transit from the axon to the soma, they come into contact with late endosomes and/or lysosomes and undergo fusion with either one of these organelles. This results in autolysosomes that become more acidic over time and are finally capable of degradation. In spite of the presence of lysosomes in the axon, they do not have the same array of degradative enzymes as lysosomes in soma [45,46]. One hypothesis is that this occurs when autophagosomes, during their retrograde travel through the axon, combine with more degradative competent lysosomes. This hypothesis has been investigated, and to some degree, it has been confirmed that preventing the retrograde transportation of autophagosomes is capable of preventing the acidification of autophagosomes and the destruction of the cargo they have ingested [47].

When autophagosomes transit to the soma, which involves input received from the axon mixed with locally produced ones, they are limited inside the soma to dendritic region and are unable to get into the axon. compartment probably makes it easier to degrade cargo by encouraging its fusion with the proteolytically active lysosomes that are concentrated in this section of the neuron [48]. Notably, lysosomes with strong proteolytic activity are concentrated in the cell body, enriching the soma for degradation. Disrupting lysosomal function has been shown to cause autophagosome accumulation only in the soma, and not in the axons or dendrites [48].

#### 2.1.2 Temporal organization of neuronal autophagy

To facilitate the maturity of the nervous system, particular factors must regulate cell division, survival, neurite extension, and synapse formation among neurons. Several lines of evidence suggest that autophagy assumes an indispensable function in neurodevelopmental disorders, and this finding lends credence to the idea that autophagy is a critical regulator to govern brain development.

Animal model studies on autophagy reveal the process's versatility in shaping the nervous system, from stimulating neurogenesis to controlling axon outgrowth and synaptogenesis. Depletion of Atg7 in embryonic primary cortical neurons and rapamycin-induced autophagy led to an increase and a decrease in neurite length, respectively [49]. Ambra1 knockout in mice result in increased neuronal proliferation, presenting failure in the closure of the neural tube as well as exencephaly [50], whereas mice whose Atg5 genes were knocked out had significantly attenuated neuronal proliferation, which was triggered by autophagy dysregulation of neurogenic signaling [51]. By promoting the turnover of signaling material carriers at leading edge of the axon or in the migratory growth cone, autophagy may contribute to axonal development. Autophagy is required for the clustering of synaptic vesicles and the development of the active zone in a *C. elegans* interneuron during the first stages of synapse formation. During neurodevelopment, autophagy may regulate inhibited axon outgrowth and appropriate synapse deposition by controlling axon outgrowth negatively and regulating the synapse-forming process positively [52].

The human genetic study also provides evidence for the role of autophagy in neurodevelopmental disorders. Elevated concentrations of the autophagy scaffolding protein WDFY3 are found in human embryonic brains that have cortical neural progenitor cells that are proliferating, providing support for the idea that this autophagy molecule performs a function in the control of prenatal neurodevelopment. Also, WDFY3 is a newly identified gene that has been linked to mild to moderate degrees of neurodevelopmental delay [53].

#### 2.1.3 Autophagy in neuronal homeostasis

Impaired autophagy has been linked to protein accumulation when one or more autophagy genes in neurons are downregulated, as well as to neurodegenerative diseases, illustrating that autophagy assumes a crucial function in maintaining brain cell viability and sustaining proper neuronal homeostasis. For example, axonal swelling in many brain areas, such as the cerebral cortex, the hippocampal area, and the nucleus gracilis, as well as the loss of Purkinje and cerebral cortex pyramidal neurons, were the results of conditional knock-out of Atg5 in mice neural progenitors [51]. Similarly, Mice deficient in Atg7 in the brain displayed abnormal behavior, suggesting that its loss causes neurodegeneration, as evidenced by features, such as less coordinated movement and aberrant limb-clasping responses. Atg7 depletion produced severe neuronal loss in the cerebral and cerebellar cortical areas, leading to the death of these mice by the time they were 28 weeks old [54].

Autophagy in neurons performs a crucial role in preventing axonal degeneration and preserving homeostasis at axon terminals. Research results demonstrate that specific ablation of Atg7 in Purkinje cells initially causes cell-autonomous, progressive axonal swellings and degeneration of the axon terminals [55]. Moreover, existing genetic evidence suggests that FIP200 has a role in regulating neuronal homeostasis via the function it plays in the autophagic process in vivo [56].

The findings from human studies suggest autophagy is also involved in neural homeostasis. Rare neurodevelopmental disorders in humans caused by missense mutations in WDR81 comprise disequilibrium syndrome, mental retardation, and cerebellar ataxia [57]. Researchers discovered elevated levels of LC3-II and beclin in the brains of people with Lewy body dementia, indicating that alpha-syn is targeted for removal by macroautophagy and that oligomeric or mutant forms are more vulnerable to this process. Autophagy was inhibited, leading to an enhancement in the accumulation of potentially pathogenic oligomers of alpha-synuclein, This provides credence to the hypothesis that neuron autophagy is necessary for maintaining protein homeostatic balance and has both pathological and therapeutic significance for synucleinopathy as well as Parkinson's disease, determining the circumstances in which autophagy would not be adequate to break down alpha-syn aggregates [58,59].

#### 2.1.4 Autophagy in neuronal activity and plasticity

Autophagy also emerged as a regulator of neuronal activity and plasticity for its established role in presynaptic compartments and synaptic activity. Synaptic connections are the fundamental building block for a variety of neuronal actions and brain functions and are responsible for the generation of precise neural circuits, whose neuronal plasticity is carried out primarily via the functional and structural alteration of synapses. Additionally, for brain plasticity to be possible, the processes of protein production and breakdown need to be strictly regulated in both a temporal and spatial way [60,61]. Imaging methods with a higher resolution, like super-resolution photography, have shown that the synapse is extraordinarily densely concentrated with proteins and modifying synaptic structure, plasticity, and function may be accomplished by regulation of autophagy when the quantity of certain synaptic proteins is altered in a temporal and spatial context [62,63]. Researchers' attention has been drawn, over many years, to the proteolytic function that the ubiquitin-proteasome system (UPS) and the endosomal-lysosomal system perform in neuronal plasticity and memory and discovered that the UPS is responsible for the regulation of pre-and post-synaptic proteins, which are both essential for neurotransmission as well as synaptic plasticity [64,65]. Indeed, Liang developed a conceptual framework to explain how the breakdown of synaptic subunits caused by autophagy can influence synaptic transmission and promote synaptic plasticity.

In particular, the activation of autophagy has been favorably linked to the improvement of synaptic plasticity deficiencies and cognitive dysfunction at the cellular and organismal level in several investigations involving rodents. Autophagy may be involved with bidirectional synaptic plasticity deficits generated by prenatal stress in adolescent male-offspring since autophagy antagonists dramatically abolished the abnormalities in gene expression triggered by corticosterone [66]. Moreover, In the hippocampus of hindlimb-unloaded mice and melamine rats, rapamycin produced a discernible increase in the total superoxide dismutase activity while simultaneously producing a remarkable reduction in the levels of reactive oxygen species [67]. These results show that increasing autophagy may partly alleviate the hippocampal synaptic plasticity deficits and the regulation of autophagy has the potential to become a novel target therapy for the management of melamine-induced synaptic plasticity deficits.

Collectively, the findings of these studies point to the significance of the autophagic pathway in neural functioning and plasticity. Furthermore, the degrading of synaptic subunits by autophagy may affect synaptic transmission and promote synaptic plasticity.

### 2.2 Glial autophagy

A complex network of intercellular connections is established and maintained by glias, which are clumps of cells present in the brain that are distinct from neurons. Glias are essential for brain growth, metabolism, synaptic function, and healing after damage. They make up about half of all the cells in the human brain and demonstrate a significant deal of heterogeneity among the various areas of the brain [[68], [69], [70]]. Glia cells are not directly involved in the transmission of electrical signals or the processing of information; however, they do replicate and react more forcefully to shifts in nutrient levels, demonstrating characteristics that are similar to those of peripheral cells but distinct from those of neurons [71,72]. During neurodevelopment and neuroprotection, autophagy plays a crucial function in glia as well as in neurons. Glias are also crucial for nutritional support, the establishment of the blood-brain barrier, the maintenance of extracellular ion homeostasis, and synaptic remodeling [73,74]. Glial cells are generally classified into microglia, astrocytes, and oligodendrocytes [75,76].

#### 2.2.1 Microglial autophagy

The immune cells distributed in the brain are referred to as microglia, and they constitute up to 10 % of the total population of the central nervous system (CNS) and perform a vital role as the brain's immune surveyors in the process of protecting against nervous disorders and preserving the equilibrium of the CNS during the many phases of growth and development. The impact of microglial autophagy on brain function is the subject of an increasing number of research papers. Autophagy in microglia has a role in synaptic pruning and myelination, both of which are important for maintaining the homeostasis of the CNS [[77], [78], [79]]. Furthermore, autophagy in microglia is implicated in several neurological disorders. According to research conducted utilizing Atg7 knockout mice and cell cultures, microglia autophagy was discovered to have a unique function in the elimination of extracellular β-amyloid (Aβ) fibrils and regulation of the Aβ-elicited NLRP3 inflammasome, which were vital for Alzheimer's disease (AD) progression [80,81]. Research shows that a particular disruption of non-canonical autophagy in microglia might diminish the ability to remove beta-amyloid and speed up neurodegeneration in a mouse model of Alzheimer's disease [81]. In Parkinson's disease (PD), an aberrant accumulation of α-Syn, which is a critical hallmark of the disease, triggers the stimulation of microglia, which, in response, enhances the pathological accumulation of α-Syn in the substantia nigra dopamine neurons [82,83]. Microglial autophagy has been linked to the breakdown of α-Syn as well as the blocking of NLRP3 inflammasomes, both of which are important in reducing neuroinflammation and preserving homeostasis in the brain [84]. Peng et al. propose that microglia autophagy act as a “brake and accelerator” pedal in ischemic inflammation for its regulation function on inflammatory responses in ischemic stroke in both negative and positive ways [85]. Serials of reviews summarized the relationship between microglial autophagy and neurological diseases, including PD, AD, and stroke [78,[85], [86], [87]].

#### 2.2.2 Astroglial autophagy

Astrocytes are the most pervasive kind of cell found in mammalian brains and may make up about 40 percent of the total number of brain cells [88]. Homeostasis in the brain is largely dependent on astrocytes, which play a crucial regulatory role in this process and are responsible for various essential processes that are linked to neurological diseases, including the transport and recycling of neurotransmitters, the metabolism of nutrients and ions, and the protection of cells from oxidative stress. Researchers are investigating what role autophagy-related proteins and genes play in astrocytes. The formation of astrocytes in the mouse brain may be regulated by Atg5, and a lack of Atg5 may inhibit the creation of astrocytes in vitro and in vivo [89]. An additional autophagy activator gene known as ULK2 is involved in the inhibition of the transformation of astrocytes in vitro [90]. The process of autophagy in astrocytes has been shown to have a variety of roles in the functioning of neurons. On the one hand, autophagy in astrocytes clears unwanted or misfolded proteins. It was proposed by Shira Simonovitch that ApoE4, which inhibits autophagy in astrocytes, is linked to a decreased ability to remove Aβ plaques in Alzheimer's disease. Autophagy in astrocytes is also critical for regulating the pathogenic consequences of ApoE4 [91]. overexpression of autophagy in astrocytes could modify the levels of a-synuclein and mitigate the toxicity imparted by the aggregation of a-synuclein on astrocytic and neuronal cells [92,93]. Conversely, during neurological diseases, autophagy in astrocytes acts to control the inflammatory process. Autophagy dysfunction in astrocytes is a crucial factor in the development of amyloid beta-induced neuroinflammatory reactions, and increasing autophagy may attenuate the inﬂammation response generated by amyloid beta, demonstrating a neuroprotective mechanism [94]. As per the findings, inhibiting LRRK2 kinase activity may cause an increase in the level of autophagy that occurs in astrocytes, one of the most important genetic variables that have been linked to the onset and progression of PD [95,96].

### 2.3 Neurovascular autophagy

In the brain, astrocytic end-feet surround the vast majority of blood vessels, constituting the neurovascular unit composed mostly of astrocytes, pericytes, neurons, and endothelial cells. Neurovascular regulation creates a steady state conducive to healthy brain growth [[97], [98], [99]]. Due to its crucial role in preserving vessel function, autophagy is of paramount significance to neurovascular units, which play a dynamic role in pathological processes of acute cerebral infarction. Since the neurovascular unit presents as a potential target for treating stroke, Zhang explored the impact of autophagy induced by ischemia damage on the neurovascular unit as a therapeutic target for stroke. They found that oxygen-glucose deprivation could induce the over-activation of autophagy, which augments the apoptotic activity in neurovascular units [100]. regulation of mitochondrial autophagy by 14,15-epoxyeicosatrienoic acid via the SIRT1/FOXO3a signaling pathway protected against glucose deficiency and reperfusion-mediated cell death in cerebral microvascular endothelial cells, which is a constituent of the neurovascular unit and has a prompt and dynamic response to acute ischemia [101]. Autophagy is an essential component that participates in nano-alumina (also known as aluminum oxide nanoparticles, is a form of aluminum oxide that is engineered at the nanometer scale) mediated neurovascular toxic effects in the CNS. When nanoalumina is administered systemically, there is an increase in autophagy-associated genes as well as autophagy processes in the brain. Additionally, there is an increase in the blood-brain barrier's (BBB) permeability [102]. Furthermore, mice who had a localized ischemic stroke model were shown to have a greater brain infarction volume after being exposed to nanoalumina. Cystatin C has been postulated as a therapeutic mediator against PD due to its ability to enhance neuron autophagy in neurovascular units [103].

## 3 Exosomes

Extracellular vesicles (EVs) is an umbrella term for a series of membrane-limited nanoparticles secreted from living cells, comprising various types of vesicles such as microvesicles and ectosomes, both of which originate from the plasma membrane, as well as apoptotic bodies [104]. Exosomes are a type of EVs that have garnered a lot of interest for the crucial functions that they play in establishing promoting metastasis and establishing metastatic. The term “exosome” was coined in 1981 after discovering neoplastic cell line-derived exfoliated vesicles that reflected the 50-nucleotidase function of the progenitor cells [105]. According to the International Society for Extracellular Vesicles (ISEV) definition criteria, 'exosomes' are a specific subtype of extracellular vesicles (EVs) derived from the endocytic pathway, typically ranging in size from 30 to 120 nm [[106], [107], [108]]. However, it is important to note that distinguishing between exosomes and vesicles of similar size and density that are derived directly from the plasma membrane is challenging. For the sake of simplicity and consistency with the articles reviewed, the term “exosome” will be used to refer to these small extracellular vesicles in the size range of 30–120 nm throughout this review.

Numerous kinds of cells are responsible for the production of exosomes, which are first thought of as waste-storing containers that are produced during the maturation of reticulocytes. However, the regulatory role of intercellular communicators that are responsible for the transportation of various proteins and nucleic acids is beginning to become clear. Exosomes are present in practically all body fluids, including blood plasma and serum, milk, amniotic fluid, semen, cerebral fluid, synovial fluid, urine, and cell culture media [109]. After being delivered to recipient cells, the signaling proteins, miRNA, mRNA, DNA, and lipids that are included in exosomes can alter the recipient cells' biological processes [110,111].

The formation of intraluminal vesicles (ILV) within endosomal compartments is the first step in the biogenesis of exosomes, beginning with the invagination of endosomal membranes. During endocytosis, the plasma membrane invaginates and pinches off to form early endosomes. These early endosomes can subsequently fuse with other smaller vesicles that originated from similar membrane invaginations and scission processes, such as clathrin-coated vesicles or caveolae. Early endosomes go through a process known as endosome maturation, in which they are subjected to several metabolic changes that eventually result in the formation of late endosomes, which then combine with lysosomes. Several endosomes go through an additional membrane invagination and fission process while they are maturing, which results in the production of a large number of ILVs inside the endosomal compartments, promoting the creation of multivesicular bodies (MVBs) as a consequence. The vast majority of MVBs fuse with lysosomes to facilitate the destruction of cargo, whilst others travel to the plasma membrane, where they fuse with the membrane and are then released into the extracellular environment as exosomes [112]. Endosomal sorting complex required for transport (ESCRT)-dependent pathway is the most frequently studied mechanism for exosome formation. A ubiquitinated pathway is mediated by the ESCRT complex. This pathway recognizes and maintains ubiquitinated proteins that have been designated for packing in the late endosomal membrane. There are four distinct complexes within the ESCRT: First, ESCRT-0 organizes cargo into clusters, and then ESCRT-1 is brought to the endosomal membrane; Membrane buds are formed by ESCRT-I and ESCRT-II; and ESCRT-III triggers the severing and releasing of vesicles. Spiral structures of ESCRT III are thought to restrict the ILV neck, whereas the ATPase VPS4 is accountable for the membrane scission [113,114]. This leads to the production of MVBs, which may subsequently fuse with the plasma membrane of the cell and discharge the ILVs into the extracellular environment in the form of exosomes. Although ESCRT is the most described machinery, exosome biogenesis may also occur through many other mechanisms that do not involve ESCRT [115].

### 3.1 Exosomes on neurological function (Fig. 3)

#### 3.1.1 Exosomes regulate inflammation in the brain

In response to pathogenic and/or viral assaults, the nervous system defends itself via a process of neuroinflammation, which includes the inflammatory stimulation of astrocytes, microglia, and glial cells. Excessive and uncontrolled neuroinflammation can cause secondary tissue damage in addition to primary brain injury, which can then progress to neuronal injury and dysfunction and appear as a diverse collection of neurological symptoms [116]. The study also indicated that neuroinflammation can have protective outcomes for the neural system, including the protection of components of the CNS, the proliferation and maturity of various neural precursor populations, axonal regeneration, and the reformation of myelin on denuded axons [117,118].

Exosome-elicited inflammatory reactions are gaining interest and are thought to be a prospective therapeutic target among the several anti-inflammatory treatments that are currently available for the treatment of neurological diseases. MiR-542-3p, an miRNA carried by exosomes, is produced from mesenchymal stem cells that blocks inflammatory reactions and protects against cerebral infarction, proposing potential therapeutic approaches for treating cerebral ischemia damage by utilizing exosome transportation of miR-542-3p [119]. PDE4B was the target of exosomal miR-124-3p, which attenuates the activity of the mTOR signaling pathway and its downstream targets p-4E-BP1 and p-P70S6K, thereby promoting neurite outgrowth after scratch injury, enhancing the outcome of neurologic conditions, and inhibiting neuronal inflammation in mice with repetitive traumatic brain injury (rTBI). The findings demonstrated that elevated miR-124-3p levels in microglial exosomes post-TBI could inhibit neuroinflammation and play a role in neurite outgrowth when they are transferred into neurons. Furthermore, microglial exosomes that have been modified using miRNAs could provide a novel treatment for TBI [120]. Exosomes have a role in PD as mediators of neuroinflammation, and exosomes that carry miRNA have the potential to be diagnostically useful as biomarkers for the disease. Immune cells that are involved in PD have the potential to actively produce exosomes in response to specific stimuli from exosomes or after being stimulated by other factors. The pathophysiologic condition of the cells from which exosomes are released is strongly linked to the exosome composition that regulates cell function and gene expression. Therefore, the exosomes in the blood or CSF that are recovered from PD patients at various stages of the disease might function as possible biological markers of the progression of the disease [121,122]. Researchers Rajendran and colleagues discovered that exosomes were responsible for the secretion of a component of the amyloid beta protein and that proteins unique to exosomes were discovered to be enriched in the amyloid plaques of AD patients [123]. Additional research has shown that AD patients' brain-derived exosomes have elevated levels of the protein amyloid beta, which is a primary constituent of the senile plaques that are diagnostic of AD and that inhibiting the absorption or release of these exosomes in vitro may inhibit the propagation of these aggregates and the subsequent cytotoxicity [124].

#### 3.1.2 Exosomes affect neurostructural integrity in the brain

Maintaining functional connections between neurons necessitates sustaining neurostructural integrity as a primary concern. Exosomes affect neurological function through various neurostructural integrity processes, including neurogenesis, migration, neurite growth, and synaptogenesis.

In the primary cortical neuronal scratch model, elevated miR-124-3p levels found in microglial exosomes post-TBI may lead to an increase in both the total count and total length of neuronal branches and inhibit the activity of mTOR signaling once they have been transferred into neurons, which in turn contributes to the outgrowth of neurites. This demonstrates that microglial exosomes that have been altered using miRNAs might offer a potential treatment for TBI as well as other neurologic diseases [120]. Moreover, a team from China reported that exosomes generated from M2 microglia contain a miR-124, which protects neurons from axonal injury and promote neurite growth after oxygen-glucose depletion by inhibiting the expression of E3 protein ligase RNF38 and promoting the secretion of neuronal regeneration-related protein (NREP) [125]. The progressive and widespread acceptance of the crucial function that the glial scar plays in neurology has emerged. Glial scarring, caused in part by reactive astrocytes, may inhibit axonal regrowth [126]. Astrocyte growth was stifled by miR-124, which was secreted in tiny extracellular vesicles by M2 microglia after stroke and increased neural axon regrowth by inhibiting glial fibrillary acidic protein (GFAP) and signal transducer and activator of transcription 3 (STAT3), thus aiding in neurological rehabilitation [127].

Small extracellular vesicles secreted by M2 microglia constitute miR-124, which may inhibit Notch1 and promote Sox2 expression levels in astrocytes, thereby promoting neurogenesis after stroke [127]. That exosomes communicate across cells in developing brain circuits to enhance neurogenesis and neural circuit growth and functionality, and that exosomes carry a varied protein payload that affects numerous performance indicators of neurological development. When administered systemically, Yang et al. discovered that exosomes with rabies virus glycoprotein (RVG) linked to exosomal protein lysosome-associated membrane glycoprotein 2b (Lamp2b) effectively delivered miR-124 to the infarction location and facilitate neural progenitors in the cortex to obtain neuronal identity and protect against ischemic injury by robust cortical neurogenesis [128]. In depressive disorders, miR-146a-5p is transferred from microglia to neurons through exosomes. The overexpression of miR-146a-5p in the DG of the hippocampus region inhibits neurogenesis while adult neurogenesis deficiencies and depressive-like behaviors may be alleviated in rats by downregulating the expression of the miR-146a-5p gene [129]. Exosomal miR-139-5p levels were discovered to be considerably elevated in the blood and brains of mice that had been genetically engineered to have a major depressive disorder (MDD). Exosome therapy and treatment with miR-139-5p antagomir both resulted in enhanced hippocampus neurodevelopment in mice that had been subjected to unpredictable moderate stress and treating normal mice with exosomes derived from patients with MDD resulted in attenuation in hippocampus neurodevelopment [130]. From these findings, exosomal microRNAs can potentially operate as a crucial regulator of neuronal development under circumstances of pathophysiological conditions caused by depression and exosomal microRNAs have a great deal of potential as diagnostic and therapeutic targets for depressive disorders.

Myelin loss limits neurological recovery and myelin regeneration and exosome aggregation may restore myelin sheath functioning. Exosomes rich in the miR-17-92 cluster have the potential to improve axon-myelin remodeling, which in turn might lead to improved motor electrophysiology and functional improvement after stroke [131]. Electroconductive hydrogels have electrical and mechanical characteristics that are identical to those of brain tissue. The combination of electroconductive hydrogels with exosomes results in a synergistic enhancement of myelinated axon development and promotes neuronal regeneration [132].

#### 3.1.3 Exosomes affect the integrity of the blood-brain barrier (BBB)

The BBB is a robust interface between the blood and brain tissue that strictly controls brain homeostasis by electively preventing substances from passing through. Numerous neurodegenerative diseases have been linked to BBB permeability. In light of exosomes' contribution to the development and progression of neurodegeneration, the BBB-crossing capacity of CNS exosomes has sparked interest in their evaluation as candidate biological markers.

The exosomes' role in mediating cell-to-cell crosstalk has, at least to some degree, a bearing on whether or not the BBB phenotype may be regulated or influenced. An adherens junctions-associated protein's level of expression may be modulated in a zebrafish model by EXOs obtained from the brain that contains the miR-132 and increased permeability of the BBB was caused by exosome inhibition that targeted miR-132 [133]. Poststroke rats pretreated with exosomes generated from serum showed dramatically decreased infarct sizes and improved neurological function, according to a research report on the protective effects of exosomes extracted from healthy rat serum in preventing ischemic stroke, revealing that healthy serum exosomes protected neurons from damage caused by experimental stroke via the mechanism of suppressing apoptosis in endothelial cells and the collapse of the BBB [134].

The exosomes released from brain capillary endothelial cells perform an instrumental function in the bidirectional signaling that helps control BBB permeability. The natural ability of exosomes to cross the blood-brain barrier can be combined with noninvasive and highly effective administration emerging as efficient delivery agents of therapeutics and molecular information to the brain, which provides a novel perspective on the field of neurology and psychiatry. For example, research has developed a technique for the monitoring of exosomes in vivo that does not need any kind of intrusive procedure. The glucose transporter GLUT-1 is responsible for the absorption of the glucose-coated gold nanoparticles (GNP) that were utilized to label the EXOs. Furthermore, enhanced aggregation at the lesion site was observed in a mouse model of localized brain ischemia after intranasally delivered GNP-labeled exosomes were noninvasively monitored. The authors found that taking this approach may help forward the investigation and use of EXOs-based therapeutics for neurological disorders. The authors argue this method is useful for diagnosing a wide variety of neurological conditions and possibly enhances neuronal recovery treatments based on exosomes [135]. As per a summary provided by Katakowski et al., exosomes generated from dendritic cells have been employed extensively as medication delivery carriers in the treatment of primary brain tumors [136].

#### 3.1.4 Exosomes inhibit apoptosis in the brain

Apoptosis, a highly conserved programmed cellular death process, is critical to the normal development and homeostasis of multicellular organisms. Neuronal apoptosis is a contributing factor in both ischemic brain injury and neurodegenerative diseases. Caspase activation is an essential event in the apoptosis signalling pathway. Exosomes generated by MSCs have been extensively investigated for their ability to regulate apoptosis in neural cells. For example, exosomes obtained from bone marrow MSCs enriched with miR-146a-5p might confer neuroprotective properties and functional benefits following intracerebral haemorrhage by lowering the neuronal apoptotic rate. In vitro studies have shown that exosomal miR-223, produced by MSCs, suppresses neuronal apoptosis by targeting PTEN. Additionally, exosomes recovered from the serum of patients with AD enhance cell apoptosis, suggesting that exosomal miR-223 generated by MSCs protects neurons from apoptosis and presents a viable treatment method for AD [137,138]. Carl Randall Harrell further evaluated exosomes generated from MSC as a potential innovative therapeutic for treating neurocognitive disorders. One of the essential roles of these exosomes is attenuating the apoptotic loss rate that occurs in brain cells [139]. Exosomes have been shown to reduce the severity of brain damage caused by ischemia/reperfusion by inhibiting apoptosis in microglia. Exosomes produced from M2 microglia were shown to minimize the level of neuronal death caused by oxygen-glucose deprivation, thus attenuating ischemic brain injury and promoting neuronal survival. The miR-451a present in exosomes exert a neuroprotective function against cerebral ischemia and reperfusion damage by reducing associated inflammatory responses, oxidative stress, and apoptotic [140]. Therefore, restoring lost neurological function may be possible via the inhibition of apoptosis.

## 4 Cross-regulation of autophagy and exosomes

Autophagy and exosome secretion play multifaceted roles in maintaining cellular homeostasis, contributing to the regulation of proteins, RNA, and membranes within cells. The understanding of their connection and interplay has garnered significant interest in recent years. There is a well-defined antagonistic relationship between autophagy and exosome production during amphisome disintegration. Furthermore, both autophagic degradation and exosome secretion are key mechanisms in the elimination of protein aggregates in neuronal cells. MVBs represent the most direct link between the exosome biosynthesis pathway and autophagy. These MVBs can be directed towards one of two different pathways [141]. On the one hand, following the activation of autophagy, autophagosomes and MVBs might combine to produce amphisomes, which then merge with lysosomes for degradation. In this scenario, MVBs are unable to merge with the neuron membrane, leading to the activation of autophagy and a decrease in exosome production. On the other hand, inhibition of autophagy has been shown to restore exosome production. The free fusion of MVBs with the plasma membrane and the subsequent release of exosomes into the extracellular milieu is characteristic of an autophagy-deficient state; however, MVBs do not merge with autophagosomes to form amphisomes [[142], [143], [144]].

Autophagy and exosomes share a common molecular basis. First, both processes originate within the canonical endocytic pathway. As previously mentioned, shared molecular mediators are present throughout autophagic and exosome processes, allowing MVBs to merge with lysosomes for cargo degradation. Moreover, endocytic vesicles transform into early endosomes and eventually into MVBs. When MVBs merge with the cellular membrane, they release ILVs into the extracellular environment as exosomes. Secondly, autophagy-related proteins directly mediate the link between autophagy and exosomes. Atg, a typical hallmark of autophagy, is extensively involved in exosome biogenesis. The Atg12- Atg3 complex, essential for the final stage of autophagosome synthesis, interacts with ALIX, a protein associated with the ESCRT mechanism, to regulate exosome development. Inhibition of Atg12- Atg3 activity reduces exosome biosynthesis and impairs late endosome transport [145]. Autophagy, regulated by mTORC1, involves a controlled degradation process that removes damaged or unnecessary cellular components for recycling, thereby maintaining metabolic homeostasis. Zou et al. comprehensively studied the regulatory function of mTORC1 in exosome release, discovering that exosome release is inhibited when mTORC1 is active and stimulated when it is inactive. Furthermore, using various experimental methods, the authors demonstrated for the first time that exosome secretion is regulated by mTORC1 and is responsive to variations in amino acid and growth factor conditions [146]. Thirdly, growing evidence indicates that cargoes contained in exosomes are implicated in the autophagic flux. The detailed crosstalk between exosomes and autophagy will be discussed below, which are best illustrated for nervous system disease.

Many physiological stresses produce abnormal physiological substances, which can lead to various diseases. The stress response serves to protect cells from the detrimental effects of these abnormal substances. The role of the stress response is closely associated with the process of autophagy. Our team conducted a comprehensive review of the interplay between autophagy and oxidative and endoplasmic reticulum stress in the nervous system [147]. The heat shock response, a major form of stress response, also plays a crucial role in the regulation of autophagy. Heat shock stress has been demonstrated to induce autophagy in neuroblastoma, cardiomyocytes, and hepatocellular carcinoma [148,149]. Various heat shock proteins are involved in the activation of autophagy. For example, the common small heat shock protein HSPB8 can recognise deleterious proteins and guide them towards autophagic elimination. The downregulation of HSPB8 and BAG3 reduces the phosphorylation level of p62 [150,151]. Heat shock factor 1 (HSF1), a master regulator of the heat shock response, contributes to the expression of autophagy-related factors such as Atg7 and Atg4B. These findings suggest a collaborative effort between stress response pathways and autophagic processes in maintaining protein homeostasis within the body [[152], [153], [154]]. Furthermore, EVs, including exosomes, which transport heat shock proteins, have emerged as a significant area of investigation in human pathology. Studies conducted over the past decade have shown that specific conditions, as opposed to physiological circumstances, influence how EV-producing cells affect the release of EVs containing heat shock proteins, thereby impacting the abundance of these proteins [[155], [156], [157]]. Therefore, although direct evidence is lacking, it is reasonable to infer that exosomes harbouring heat shock proteins may contribute to the progression of the autophagic response under particular environments and conditions [149], thereby regulating physiological functions such as the removal of harmful proteins and the maintenance of physiological balance.

### 4.1 Exosomes regulate autophagy in brain diseases (Table 2)

#### 4.1.1 Alzheimer's disease (AD)

AD is an irreversible, age-associated neurodegenerative disorder that is characterized by progressive learning and memory deficits. In terms of neuropathology, AD is distinguished by the aggregation of β-amyloid (Aβ) and hyper-phosphorylated Tau (tangles) [158].

There is mounting evidence that exosomes and their components contribute to the pathogenesis of AD. When studying the accumulation of APP cleavage events in MVBs, researchers discovered that AD-related exosomes, which are produced from cultured cells and contain Aβ peptides, accumulated surrounding amyloid plaques in the brains of AD patients, in which a link between exosomes and AD is observed for the first time. Exosomes bearing Aβ peptides have been shown to aggregate in and around neuritic plaques, providing additional confirmation of their involvement in the AD disease process. However, toxic Aβ and hyperphosphorylated tau are transported from cell to cell in the brain through exosomes, contributing to cell death and neuronal degeneration. The pathogenic process of AD has been linked to exosomes, and it is now hypothesized that exosomes have a double influence on AD, either worsening or enhancing the disease process. Firstly, exosomes contribute to the degenerative progression of AD by regulating the dissemination of amyloid beta and tau. Multiple experiments using animal models have demonstrated that exosomes obtained from plasma neurons of AD patients' seed tau accumulation stimulated AD-like neuropathology in normal mice CNS whereas exosomes from glial cells stimulated Aβ clumping in the brain of mice. Moreover, Aβ oligomer-laden exosomes produced from the brains of people with AD are neurotoxic. Exosomes are responsible for the secretion of tau protein from cultured primary neurons as well as from N2a cells that have overexpressed a variety of tau constructs. This release of tau protein contributes to tau transmission across synapses [159]. Secondly, exosomes might have a role in the degenerative changes of AD by stimulating neural inflammation, hampering neuronal activities, and perhaps leading to dementia [160,161]. Unlike the case with the controls, Patients having AD showed considerably reduced concentrations of cell survival factors, particularly heat-shock factor-1, low-density lipoprotein receptor-related protein 6, and transcriptional repressor element 1 (RE1) in exosomes from neurons. Conversely, AD patients exhibited considerably enhanced levels of complement, particularly pro-inflammatory factors (IL-1β, TNF-α, and IL-1) [161,162]. Inflammatory compounds in exosomes have been linked to AD, and new research suggests that complement proteins may play a role in this association. Moreover, protein regulators show individual variation at various preclinical phases of the disease, showing that the levels of complement proteins in astrocyte-derived exosomes are linked to the disease staging. Thirdly, plenty of new data shows that exosomes have a preventive function in AD, in addition to their potential detrimental consequences. Cystatin C, neprilysin (NEP), and insulin-degrading enzymes are among the many compounds found in exosomes that have been shown to have protective properties against AD, which play a role in clearing away Aβ so that neuronal functioning may be restored [163,164]. Simon et al. postulated that secreted tau, which is secreted via exosomes mediated by tau overexpression, does not trigger cell death and could attenuate the toxicity resulting from excessive tau in cells [165]. Additionally, In vitro studies have shown that exosome production may prevent Aβ oligomerization by increasing the amount of Aβ that is cleared by microglia, however, exosomes decrease Aβ and amyloid depositions in APP-transgenic mice [166,167].

It is now well accepted that the crucial function that exosomes play in the degenerative process of AD is strongly linked to regulating autophagy. Wu et al. [168] investigated the role that exosomes produced from astrocytes with cholesterol accumulation played in the pathogenesis of AD and discovered that after U18666A treatment, generated exosomes from astrocytes produce an increment of autophagy marker LC3-II, a lysosomal marker of cellular lysosomal-associated membrane protein 1 (LAMP1), and cathepsin D. These data, together with earlier ones, show that exosomes produced from astrocytes showing cholesterol accumulation may assume an essential function in the transportation of APP/Aβ peptides and also in impacting neuronal survival in the brains of people with AD.

BACE1, which stands for beta-site amyloid precursor protein cleaving enzyme, is capable of cleaving consecutive APP into Aβ. Therefore, BACE1 is significantly involved in the process through which AD amyloid plaques and neurofibrillary tangles are formed. Treatment with MiR-124 led to BACE1 downregulation at protein and RNA levels in addition to reducing Tau but not APP via the regulation of the autophagy pathway for reversing the over-expressing autophagy markers p62, Atg5, and LC3II proteins in the AD patients, and this is thought to lessen the symptoms of AD pathogenesis. Li et al. [169] found that through enhancement of PINK1/Parkin-mediated autophagy, administration of M2-EXOs has the potential to attenuate Aβ plaque formation as well as minus Aβ oligomer expression. Taking into account that miR-124 was also involved in mediating M2-EXOs' protective properties in cerebrovascular disease [170], it is plausible to speculate that miR-124, which originates from M2-EXOs, enhances PINK1/Parkin pathway-elicited autophagy to exert protective properties in the AD pathogenic mechanism. Patients suffering from AD may be distinguished from healthy case controls by the concentrations of autolysosomal proteins found in neurally generated blood exosomes, demonstrating in live individuals with AD the presence of neuronal autophagy malfunction at an early stage [162].

#### 4.1.2 Parkinson's disease (PD)

Parkinson's disease (PD) is the second most prevalent type of neurodegenerative disease globally, only after AD. The underlying pathogenesis of PD is multifactorial, including genetic and environmental, and the interplay between them, confers increased disease risk [171,172]. The presence of Lewy bodies, which are protein aggregates predominantly composed of an accumulation of alpha-synuclein (alpha-syn), is one of the most important pathological hallmarks of Parkinson's disease (PD). These entities may be seen in the brains of people with both hereditary and sporadic types of PD [173,174]. One of the most important insights into the origin and course of PD is supported by the finding that α-syn may be transmitted, thus leading to the development of more effective medicines in the future by focusing on additional molecular targets.

Exosomes are a factor in the progression of PD, and they may act in either a positive or negative manner. On the one hand, exosomes are responsible for poor neuronal function and may serve as possible intercellular transporters of pathogenic proteins and RNAs. Because α-syn oligomers are responsible for the death of neurons, the presence of α-syn in exosomes could serve as a biological marker for PD in addition to being a potentially crucial signal in the development of the neurotoxic form of α-syn and its dissemination throughout the brain. The level of alpha-synuclein found in the plasma neuronal exosomes of people with PD was substantially elevated in contrast with that seen in healthy controls [175]. On the other hand, research suggests that exosomes may have a neuroprotective function in PD. Inflammatory processes that are facilitated by exosomes are regarded as possible treatment targets for PD. In PD mice, exosomes carrying catalase produced by macrophages and monocytes attenuate inflammation in the brain and greatly boost the survival of neurons [121].

New research suggests that autophagy mediation by exosomes performs an indispensable function in the etiology of PD. Dopaminergic neurons in the substantia nigra, pancreatic β-cells, and Enteroendocrine cells in the intestine could all absorb milk exosomes carrying miRNA-148a and miRNA-21, which could then promote α-syn overexpression and inhibit autophagy, thus contributing to the onset and progression of PD [176]. Researchers led by Xia discovered that microglia had a large capacity for absorption of plasma exosomes obtained from PD patients and may be triggered by exogenous exosomes in vivo and in vitro [177]. In addition, the autophagy of the BV2 mouse microglia cell line was discovered to be dysregulated by exogenous exosomes, as evidenced by the overexpression of intracellular α-syn and an acceleration of its release into the extracellular environment. In addition, these processes may modify PD development. Autophagy-related microRNA (miRNA) was abundant in exosomes isolated from α-syn-primed MSC, and these exosomes might stimulate the production of miRNAs that regulate autophagy, enhancing neuroprotective properties in Parkinsonian models [178].

The expression of certain miRNAs helps to regulate the autophagy process, which in turn may protect against PD. Li et al. found that therapy with exosomes enriched with miR-188-3p might protect against PD by inhibiting autophagy and pyroptosis. Exosomes have the potential to be a useful therapy for PD since they may both inhibit and activate autophagy [179]. For example, autophagy induction by MSC-derived exosomes suggests their therapeutic potential in a PD model [180].

#### 4.1.3 Ischemic brain injury

It is widely established that ischemic brain injury (IBI) is a leading factor of neurological disability and diseases accompanied by severe brain dysfunction or even death all over the world, such as cognitive dysfunction, mental impairment, and possibly vegetative states. Growing evidence points to exosomes, containing multiple mRNAs, long noncoding RNAs, proteins, circular RNAs, and microRNAs, stably transfer between different cells, which are crucial for maintaining homeostasis in the brain microenvironment after IBI.

For example, the most often examined components of exosomes are microRNAs which are essential players in a variety of biochemical pathways that are implicated in IBI. Ischemic stroke patients exhibit substantially elevated plasma concentrations of exosomal microRNA-9, -15a, and −124 in contrast with healthy controls, which may prove to be useful indicators for determining the extent to which IBI has caused harm to the brain [181,182]. Exosomal concentrations of microRNA-223 and microRNA-134 are found to be considerably elevated in individuals who have had an ischemic stroke [183,184]. Furthermore, exosomal levels of microRNA-152-3p were significantly lower in individuals with ischemic stroke in contrast with control subjects [185]. According to a summary provided by Ryszard Pluta, the microRNAs 9, 124, 134, 152-3p, and 223 are linked to the severity of the ischemic stroke, whilst the microRNAs 134 and 223 are linked to a dismal prognosis of ischemic stroke [186].

The functions that exosomes play in IBI and the methods by which they regulate neuronal autophagy have become more prominent over time. Ischemic-preconditioned astrocytes (IPAS) secreted exosomes, which then served as transporters to transfer circSHOC2 to neighboring neurons, which functions in neurons as a molecular sponge for miR-7670-3p and prevent the death of neurons via the promotion of autophagy, consequently ameliorating the extent of neuron damage caused by ischemia conditions in vivo and in vitro. The findings highlight that the circSHOC2/miR-7670-3p/SIRT1 axis could constitute a therapeutic target in individuals diagnosed with IBI and might facilitate the development of an innovative treatment approach for ischemic stroke [187]. A research team from China has reported that exosomes derived from astrocytes have the ability to inhibit neuronal apoptosis and improve neuronal damage by regulating autophagy in cases of ischemic stroke. Subsequent studies have confirmed that the exosomes transfer a specific microRNA, miR-190b, which targets Atg7 to suppress autophagy [188,189]. Even though the specific circRNAs in MSC-derived exosomes that protect neurons from injury are unknown, Hu's group did find that they increased FOXO3a expression levels and hence enhanced microglial mitophagy, thereby reducing the risk of further neuronal injury, highlighting the potential for MSC-derived exosomes to serve as a novel treatment modality with promising application possibilities for alleviating newborn hypoxic-ischemic brain injury [190].

Following acute ischemic stroke, miR-30d-5p was downregulated both in patients and in animal models. A study further proved that exosomes generated from adipose-derived stem cells and enriched with microRNA-30d-5p displayed a protective function against acute ischemic stroke since these exosomes led to a considerable reduction in the region of infarction that was caused by brain damage by inhibiting autophagy and enhancing M2 polarization in microglia and macrophages [191]. Exosomes derived from adipose-derived mesenchymal stem cells also have been found to secrete miR-25-3p, which exhibits the potential to induce neuroprotection through enhancing autophagic flux in a preclinical stroke model [192]. In another rat stroke model, researchers found that exosomes that were generated from healthy serum inhibited autophagy by reverting the ratio of LC3B-II to LC3B-I, as well as preventing autolysosome production and autophagic flux, which protected neurons from an experimental stroke in part by enhancing neurological outcomes and preserving the BBB [134]. Furthermore, pigment epithelium-derived factor is involved in the protection of the brain from cerebral I/R injury since these exosomes altered the adipose-derived MSC to attenuate cerebral IR damage by regulating the apoptotic and autophagic processes [193]. Nicotinamide phosphoribosyltransferase (Nampt) is a crucial secreted protein involved in cerebral ischemia injury. Deng et al. reported that Nampt is released in astrocyte-derived exosomes, which promote neuronal autophagy by inhibiting mTOR phosphorylation and activating the AMPK pathway. This mechanism leads to the amelioration of ischemic stroke injury [194]. Heat stress-induced brain damage can result in heat stroke. In a study by Li et al. , the role of miR-155 was investigated in brain injury following heat stroke. The findings revealed that microglia can transfer miR-155 to neurons through exosomes, thereby accelerating neuronal autophagy by suppressing the mTOR pathway via targeting Rheb under heat stress conditions [195]. Neuroinflammatory responses have a significant impact on post-stroke recovery and are regulated by exosomes. Luo et al. (2023) demonstrated that HABP2 in exosomes derived from peripheral blood can activate the PAR1/PI3K/AKT/mTOR pathway, resulting in reduced autophagy and aggravated neuroinflammatory injury after ischemic stroke (IS) [196]. Recently, different exosomal contents derived from bone marrow mesenchymal stem cells (BMSCs) have shown neuroprotective effects in stroke through their role in autophagy. For instance, two groups found that long non-coding RNA KLF3 antisense RNA 1 and miR-133a-3p derived from BMSCs, respectively, exerted protective effects against cerebral ischemia injury by enhancing and inhibiting autophagy [197,198].

#### 4.1.4 Traumatic brain injury

Traumatic brain injury (TBI) is hallmarked by a neurotrauma caused by a mechanical force that is applied to the head and it is one of the most common types of brain injury causing mortality, morbidity, and economic burden globally. The pathogenesis of TBI is not just an acute occurrence but is also engaged in several cascades of biological processes in the brain that interact with one another and are dependent on one another. Several different biomarkers have been identified as candidates for the role of evaluating the severity of TBI.

Following a TBI, several recent studies have demonstrated that there is a significant shift in the exosomes and associated cargoes found in brain cells surrounding the wound. In a recent study, exosomes from traumatic brain injury patients have been shown to contain more proteins than those from healthy people [199]. Blocking the release of exosomes from the brain post-TBI may restore cognitive function, indicating that inhibiting exosome release might be a novel approach to bettering outcomes post-TBI. Recent research has shown that TBI results in persistent changes in the autophagy process [200,201]. Nonetheless, it is not yet clear if TBI improves or worsens autophagy.

Numerous research reports have shown that exosomes and the unique protein or miRNA content contained within them may effectively control autophagy to alter TBI severity and progression. Exosomes obtained from cultured BV2 microglia showed consistent elevations in miR-124-3p levels, according to the findings of one research group, which examined TBI mouse model brain extracts. Subsequent investigation found that overexpression of miR-124-3p in microglial exosomes post-TBI might serve to prevent neural autophagy and protect against nerve damage through their transport into neurons, showing that treatment using microglial exosomes enriched with miR-124-3p could constitute a unique therapeutic method for ameliorating nerve damage in patients who had had a TBI [202]. Recent research from the same group found that miR-124-3p may inhibit mTOR signaling and stimulate autophagic activation in brain microvascular endothelial cells, as a result, these cells are protected from the harm caused by the TBI [203]. miR-21-5p was shown to have an upregulated response in both in vivo and in vitro TBI models, according to the reports, and Exosomes formed from neurons and abundant in the miR-21-5p provided protective benefits by inhibiting autophagy and targeting Rab11a, Therefore, reducing the autophagy-mediated trauma-induced neuron damage in vitro following TBI [204]. Insulin-like growth factor-1 (IGF-1), is a significant hormone that performs a remarkable function in cellular growth, proliferation, and autophagy, in addition to orchestrating mitophagy by enhancing mitophagy indicators through exosomal miR-let-7e [205]. In addition, exosomes originating from bone marrow stromal cells have the potential to trigger autophagy in microglial cells through the miR-32-induced regulation of disabled homolog 2-interacting proteins, thus offering a fresh approach to the transplantation of neural stem cells in cases of brain damage [206].

#### 4.1.5 Glioblastoma

Glioblastoma (GBM) is the most common and aggressive primary brain tumor in adults, characterized by a poor prognosis and high mortality due to its pathological process, which involves a complex tumor microenvironment (TME) composed of both cancerous and non-cancerous cells. The role of autophagy in GBM is multifaceted, exhibiting both tumor-promoting and tumor-suppressing effects depending on the cellular context and TME. Autophagy can directly influence GBM cell functions, such as survival, migration, and invasion, or indirectly affect GBM by modulating the TME, including immune cell populations, tumor metabolism, and glioma stem cells [207,208]. Furthermore, autophagy intersects with other forms of cell death, such as pyroptosis and ferroptosis, highlighting a complex interplay that could inform novel therapeutic strategies for GBM [209,210]. Therefore, a comprehensive understanding of the mechanisms through which autophagy operates in GBM, as well as its interactions with other cell death pathways, is crucial for developing innovative treatment approaches.

Exosomes also play a significant role in the pathogenesis of GBM. Several studies have demonstrated the cancer-targeting capabilities of exosomes derived from GBM cells [211]. For example, research by Lee showed that U87MG-derived exosomes loaded with selumetinib (U87-Selu exo) can target GBM cells (U87MG) by exploiting their ability to home in on their parent cells [212]. Moreover, numerous studies have validated the therapeutic potential of exosomes in GBM. Sakr and colleagues, for instance, transfected glioma cell-derived exosomes with miR-150-5p or miR-133a mimics and co-cultured these exosomes with glioma cells [[212], [213], [214]]. Various chemotherapeutic agents and miRNAs have been successfully loaded into exosomes for GBM treatment. For example, Lang et al. transfected bone marrow mesenchymal stem cells with a lentiviral vector containing miR-124a, isolated the exosomes produced, and found that treating mice with intracranial glioma stem cell implants using these exosomes significantly extended their survival [215,216].

Regulation of autophagy through exosomes can significantly contribute to GBM therapy. For example, miR-21 levels in exosomes from patients with GBM have been reported to be tenfold higher than in those from control participants, suggesting that exosomal miR-21 could serve as a potential biomarker for GBM diagnosis [217]. Additionally, the regulation of autophagy is one mechanism by which exosomes influence GBM progression and treatment response. Zheng et al. demonstrated that exosomes generated by GBM stem cells contain programmed death-ligand 1, which induces autophagy via the AMPK/ULK1 signaling pathway, leading to increased temozolomide resistance. This finding highlights the potential of targeting exosome-mediated autophagy regulation to enhance the efficacy of temozolomide in GBM therapy [218].

### 4.2 Autophagy regulates the secretion and release of exosomes in brain diseases

Exosomes and autophagy have a reciprocal relationship, which is evident in autophagy's ability to regulate the secretion and release of exosomes in the functioning of the nervous system. The dysfunctional autophagy-lysosome pathway leads to impaired clearance of aberrant protein aggregates, which affects the progression of neurological disease. According to research conducted on PD, a dysfunctional autophagy-lysosome pathway may decrease the effectiveness of the removal of α-syn oligomers from the intracellular environment and increase the release of oligomers into the extracellular region, causing exosomes that contain α-syn oligomers to be passed from cell to cell and therefore accelerating the progression of the disease [219]. Activated discharge of exosomes carrying α-syn may take place as a protective mechanism in response to the obstruction of autophagy-dependent α-syn elimination. In particular, the silencing of Atg5, a key protein for autophagic vesicles, promotes the production of α-syn via exosomes, which is triggered by a reduction in cell death α-syn [220]. In addition to this, further research demonstrates that lysosomal inhibition of donor cells appeared to promote both exosome-mediated production and transference of beta-syn, demonstrating that the autophagy-lysosomal pathway is involved in the exosomal α-syn transmission [221]. Guo and colleagues showed that inhibiting autophagy flow caused a spike in the percentage of MVB-autophagosomes, resulting in a surge in the percentage of microglial exosomes that carry α-syn and the promotion of α-syn transmission through exosomes [222]. Furthermore, inhibition of autophagy caused by milk-mediated stimulation of mTORC1 and impaired autophagy may increase exosomal egress of beta-syn to the brain [176]. These findings provide credence to the premise that the progression of PD is influenced in some way by lysosomal homeostasis, autophagy, and the release of α-syn exosomes.

The fundamental cellular processes that govern how the autophagy-lysosome pathway affects the SNCA/α-syn pathway were investigated by Georgia Minakaki, and these results provided solid evidence that inhibiting autophagy facilitated the release of SNCA/α-syn and its subsequent transport through exosomes and extracellular vesicles, pointing to the idea that autophagy might influence the protein composition of exosomes and extracellular vesicles, and subsequently the progression of synucleinopathies [223].

Cazzaro et al. researched the functions that autophagy cargo receptors perform in the regulation of sEV release through pathways that alter AD development and discovered that autophagy cargo receptors, p62, and optineurin, inhibit sEV release, whose increase is mediated by AD pathogenic proteins, such as mutant tau and Aβ oligomers [224]. The findings suggest that interactions between autophagy cargos and their cargo receptors may play a role in the regulation of sEV secretion through processes that contribute to the pathophysiology of AD. Xue Zeng investigated the mechanism by which cerebral ischemia-reperfusion (IR) injury causes damage via mitochondrial injury and discovered that Drp1 (a key protein regulating mitochondrial fission) activation following cerebral IR injury enhances the development of p62-induced autophagosomes while simultaneously inhibiting the transition from autophagosomes to autolysosomes and stimulating a pathway involving RIP1/RIP3 and exosomes [225].

## 5 Conclusion and future perspectives

Significant progress has been made in understanding the roles of exosomes and autophagy in the physiology of the nervous system and the pathogenesis of neurological diseases. In recent years, emerging and intriguing research on exosome-autophagy cross-regulation mechanisms in the nervous system has garnered substantial attention and development. This review provided a brief overview of these mechanisms. As discussed herein, certain proteins involved in autophagy can influence the biogenesis and release of exosomes in the nervous system. Conversely, various substances contained within exosomes (such as RNA, DNA, proteins, and lipids) can regulate the progression of autophagy. Despite considerable strides in understanding the implications of these interactions on physiological and pathological states in the nervous system, additional comprehensive studies are warranted to provide systematic evidence.

Exosomes originating from brain cells exhibit brain-specific surface proteins and, owing to their small size, confer significant advantages in traversing the blood-brain barrier (BBB). This renders exosomes particularly attractive for targeted drug delivery to the brain, which is crucial for the diagnosis, prevention, and treatment of neurological diseases. Building upon current research on the intimate interactions between exosomes and autophagy, forthcoming endeveours can focus on the following aspects:

First, the isolation and purification of exosomes have not been standardised to date, a prerequisite for their widespread utilisation in clinical trials. Future research could leverage autophagy mechanisms as a breakthrough for improving exosome isolation and purification, thereby enhancing their potential for clinical application.

Second, more reliable biomarkers should be identified to elucidate the roles of exosomes and autophagy in neurological diseases. As previously mentioned, several biomarkers have been elucidated to play roles in exosomes and autophagy in neurological diseases, yet only a select few are deemed suitable for practical application. Future studies might combine multiple biomarkers into composite markers to optimally utilise exosomes and autophagy for the diagnosis and treatment of neurological diseases.

Third, the complexity of exosomes, particularly their potential off-target effects, poses risks to their biosafety and targeting efficacy, constituting a major challenge in the translation of exosomes into clinical practice. The regulatory role of autophagy on exosomes provides valuable insights for addressing this challenge. Through the modulation of autophagy, the composition of exosomes can be more effectively controlled, thereby enhancing their utility as therapeutic carriers and improving the acceptability of exosome-based clinical treatments.

In conclusion, future studies ought to carefully assess the precise mechanisms through which autophagy mediates different exosome secretion and release and to explore the biomedical applications of exosomes in the prevention and treatment of various brain diseases by targeting autophagy. Such studies may yield novel diagnostic, preventive, and therapeutic insights and strategies for the treatment of brain diseases.

## Data availability statement

All data are obtained in published article/supp. material/referenced during this in article.

## Funding statement

This research was funded by the 10.13039/501100001809National Natural Science Foundation of China [grant numbers 81602846; 81602846] and 10.13039/501100007129Natural Science Foundation of Shandong Province [grant numbers ZR2021MH145]. 10.13039/501100010040Taishan Scholar Project of Shandong Province [grant numbers tsqn20181215]. Science and Technology Program of Traditional Chinese Medicine of Shandong Province [grant numbers M-2022066]. Science and Technology Development Program of Traditional Chinese Medicine of Lianyungang [grant numbers YB202316].

## CRediT authorship contribution statement

**Hai-Dong Wang:** Writing – original draft, Funding acquisition. **Chao-Liang Lv:** Software, Investigation, Data curation. **Lei Feng:** Validation, Supervision, Conceptualization. **Jin-Xiu Guo:** Software, Data curation. **Shi-Yuan Zhao:** Software, Resources, Investigation. **Pei Jiang:** Funding acquisition, Conceptualization.

## Declaration of competing interest

The authors declare that they have no known competing financial interests or personal relationships that could have appeared to influence the work reported in this paper.
